# Detection of Type A Trichothecene Di-Glucosides Produced in Corn by High-Resolution Liquid Chromatography-Orbitrap Mass Spectrometry

**DOI:** 10.3390/toxins5030590

**Published:** 2013-03-22

**Authors:** Hiroyuki Nakagawa, Shigeru Sakamoto, Yuki Sago, Hitoshi Nagashima

**Affiliations:** 1 National Agriculture and Food Research Organization (NARO), National Food Research Institute, 2-1-12 Kannon-dai, Tsukuba-shi, Ibaraki 305-8642, Japan; E-Mails: sydney1219@nifty.com (Y.S.); nagasima@affrc.go.jp (H.N.); 2 ThermoFisher Scientific, C-2F, 3-9 Moriya-cho, Yokohama 221-0022, Japan; E-Mail: shigeru.sakamoto@thermofisher.com

**Keywords:** masked mycotoxin, mycotoxin glucoside, LC-Orbitrap MS, trichothecene, *Fusarium*, corn

## Abstract

The existence of di-glucosylated derivative of T-2 toxin in plant (corn powder) was confirmed for the first time in addition to that of HT-2 toxin. These masked mycotoxins (mycotoxin glucosides) were identified as T-2 toxin-di-glucoside (T2GlcGlc) and HT-2 toxin-di-glucoside (HT2GlcGlc) based on accurate mass measurements of characteristic ions and fragmentation patterns using high-resolution liquid chromatography-Orbitrap mass spectrometric (LC-Orbitrap MS) analysis. Although the absolute structure of T2GlcGlc was not clarified, two glucose molecules were suggested to be conjugated at 3-OH position in tandem when considering the structure of T-2 toxin. On the other hand, the specification of the structure seems to be more complicated in the case of HT2GlcGlc, since HT-2 toxin has two possible positions (at 3-OH and 4-OH) to be glusocylated. In addition, 15-monoacetoxyscirpenol-glucoside (MASGlc) was also detected in the identical sample.

## 1. Introduction

**Figure 1:**
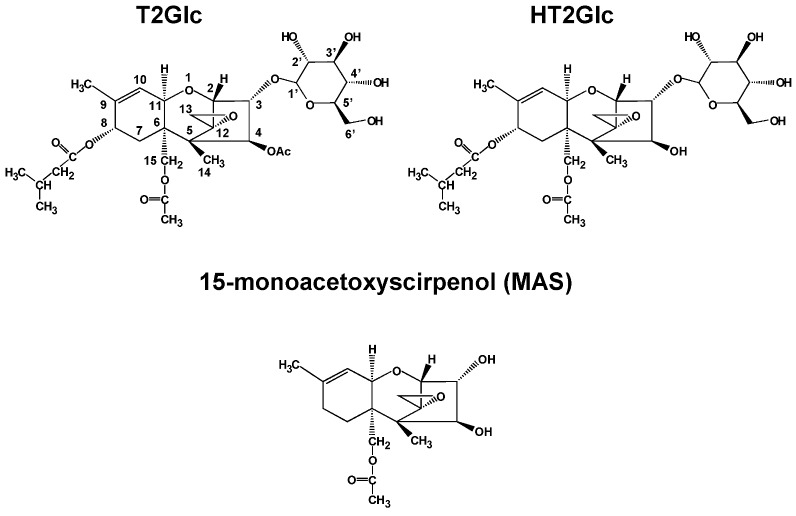
Typical structures suggested for T-2 toxin glucoside (T2Glc), HT-2 toxin glucoside (HT2Glc), and monoacetoxyscirpenol (MAS).

Corn and maize are major and important crops consumed as food and feed. Mycotoxin contamination is still prevailing as a problem concerned with them, and occasionally cause to serious sacrifice through the ingestion [1]. *Fusarium* fungi are known as plant pathogens infecting major cereals used for food and feed, and some of them produce mycotoxins such as trichothecenes, zearalenone, and fumonisins [2]. Among *Fusarium* mycotoxins, deoxynivalenol (DON), which belongs to the type B trichothecenes, is the most important [3]. Many countries set regulation values for DON [4]. Recently, a glucosylated derivative of DON, namely DON-3-glucoside (DON3Glc) was found in cereal grain and beer [5,6], and similar derivatives for other mycotoxins [7,8] were also reported. These glucoside derivatives are frequently called “masked mycotoxins”, because they are sometimes not detected by conventional analytical methods due to their higher polarity [9]. Hydrolysis of masked mycotoxins to their aglycons has also been reported [10,11,12], and it is suggested that they present an additional, potential risk to consumers. The authors previously reported the detection of masked mycotoxins not only for nivalenol, a type B trichothecene, but also for its precursor fusarenon-X in wheat grain [13]. Furthermore, the detection of those derived from the type A trichothecenes, T-2 toxin (T2) and HT-2 toxin (HT2) in corn powder [14,15], wheat, oats, and barley [16,17] as well as the cultures of *Fusarium sporotrichioides* [18] was reported. Typical structures suggested for T2-glucoside (T2Glc) and HT2-glucoside (HT2Glc) are shown in Figure 1. Among the type A trichothecenes, T2 and HT2 are the most focused due to their higher prevalence in crops, and the European Food Safety Authority (EFSA) Panel on Contaminants in the Food Chain (CONTAM) established a group of tolerable daily intake (TDI) of 100 ng kg^−1^ body weight for the sum of T2 and HT2 [19]. In this paper, di-glucoside derivatives of T2 and HT2 were detected in corn powder using a high-resolution LC-Orbitrap MS instrument. This report is the first one to have shown that T2 is possibly conjugated with two glucose molecules in plant (corn). Another di-glucoside derivative derived from HT2, which had been found in barley [17], was also discovered in corn in this study. In addition, a mono-glucoside derivative of 15-monoacetoxyscirpenol (MAS) whose structure is shown in Figure 1, was also found in the identical sample.

## 2. Materials and Methods

### 2.1. Chemicals

T2 (*MW* = 466.2197) and DON were purchased from Wako pure chemical Industries Ltd. (Osaka, Japan). HT2 (*MW* = 424.2092) and MAS (*MW* = 324.1567) were purchased from Sigma-Aldrich Co. (St. Louis, MO, USA). DON3Glc was obtained from Wako as a standard solution (50 mg L^−1^) in acetonitrile. Acetonitrile (LCMS grade) was from Wako, and distilled water (LCMS grade) was obtained from Kanto Chemical (Tokyo, Japan). Ammonium acetate (chemically pure grade) was from Kanto, and acetic acid (>99.9% of chemically pure grade, not glacial) was from Wako. All other chemicals used were commercially available and of a chemically pure grade.

### 2.2. Corn Powder Sample

Mycotoxin reference material of corn powder (batch number MTC-9999D) from Trilogy Co. Ltd (Washington, DC, USA) was used as previously reported [15]. The material was contaminated with T2, HT2, and DON. The manufacturer-labeled concentrations of the toxins were 300 ± 57 μg kg^−1^ (T2), 510 ± 83 μg kg^−1^ (HT2), and 2200 μg kg^−1^ (DON). It was stored at −20 °C in the dark until analysis.

### 2.3. Stock Solution and Working Solution Preparation

T2, HT2, MAS and DON were weighed accurately, individually dissolved in acetonitrile, and the volumes of these solvents were adjusted so that their concentrations were 100–200 mg L^−1^. These stock solutions were stored in brown glass containers at 4 °C to prevent photo-degradation and evaporation of the mycotoxins. For the working solutions, each stock solution was diluted with acetonitrile/water/acetic acid (5/94/1, *v*/*v*/*v*).

### 2.4. Extraction and Purification of Mycotoxins

Extraction and purification were performed as formerly reported [15]. Corn powder (10 g), 40 mL of acetonitrile/water (80/20, *v*/*v*), and 0.4 mL of acetic acid (>99.9%) were homogenized, centrifuged at 2000*g* for 10 min, and a portion of the supernatant (7 mL) was loaded directly on a Bond Elut Mycotoxin column (Agilent Technologies, Santa Clara, CA, USA, Part No.12165001B) for clean up. After discarding the first 3 mL of solvent coming off the column, a 1.6 mL aliquot of the remainder was dried under a nitrogen gas stream at 40 °C. The residue was re-dissolved in 0.2 mL of acetonitrile/water/acetic acid (5/94/1, *v*/*v*/*v*) for LC-MS analysis.

### 2.5. LC-MS Analysis

Detection and identification of the masked mycotoxins was performed with a LC-Orbitrap MS “Exactive” (ThermoFisher Scientific, Waltham, MA, USA) according to the similar procedure as reported by Nakagawa *et al*. [13,15]. Chromatographic separation was performed as previously reported [13] using a HyPurity C_18_ column (250 × 3 mm i.d., 5 μm particle size) (ThermoFisher Scientific) with the HPLC system composed of Accela pump and Thermo PAL auto injector (ThermoFisher Scientific). The system was operated in the positive [15] or negative [13] polarity in the mass range of *m/z* 70–1000, at the resolving power of 100,000 full width at half maximum (FWHM) (*m/z* 200). At each polarity, the accurate mass/high resolution (AM/HR) full scan (scan event 1) and all ion fragmentation MS/MS scan with HCD (higher energy collision induced dissociation) energy of 17 eV (scan event 2) were concurrently performed in a single run. Although precursor ion isolation is not achieved by “Exactive”, the consistency of the retention time of the precursor (observed with scan event 1) and reliable fragments (observed with scan event(s) 1 and/or 2) guarantees that those precursor and fragments should be related. For the mass accuracy estimation, the mass value observed as an abundant ion extracted at the apex of the chromatographic peak was used. The exact mass values (calculated and observed) of the analytes’ ions are summarized in Table 1, Table 2, Table 3. In accordance with the European Commission guideline [20], mass deviation <5 ppm from the calculated value was used as the criterion for compound identification. Since the neutral losses of isovaleric acid (C_5_H_10_O_2_), acetic acid (C_2_H_4_O_2_), and formaldehyde (CH_2_O) were confirmed to be characteristic for T2Glc and HT2Glc [15], such losses were equally examined for T2GlcGlc, HT2GlcGlc, and MASGlc as listed in Table 1, Table 2, Table 3. Working solutions containing T2, HT2, MAS, and DON at concentrations between 10 and 1000 ppb were prepared, and used for calibration. For the detection of T2, the exact mass value calculated in the form of [M + NH_4_]^+^ adduct (484.2541) was used as monitor ions. For the detection of HT2, MAS, and DON, the scan event was performed in the negative polarity, and the exact mass values calculated in the form of [M + CH_3_COO]^−^ adduct (483.2236, 383.1711 and 355.1398, respectively) were used as monitor ions. Because analytical standards for T2GlcGlc, HT2GlcGlc, and MASGlc were not available, their amounts in corn powder could not be quantified.

**Table 1:** Exact mass values of T2GlcGlc and relative ions (calculated and observed) at positive polarity.

Elemental formula	Ion	Calculated mass ^a^	T2GlcGlc (12.94 min)	T2Glc (13.45 min)	T2 (15.85 min)
			Observed mass ^b^ (mass accuracy, ppm)	Observed mass ^b^ (mass accuracy, ppm)	Observed mass ^b^ (mass accuracy, ppm)
C_36_H_58_O_19_N	[T2GlcGlc + NH_4_]^+^	808.3598	N.D ^c^	-	-
C_36_H_54_O_19_	[T2GlcGlc + H]^+^	791.3332	791.3297 (−4.49)	-	-
-	-	-	791.3300 ^d^ (−4.11)	-	-
C_30_H_44_O_14_	[T2GlcGlc–Glc + H]^+^ ([T2Glc + H]^+^)	629.2804	N.D ^c^	629.2792 (−1.91)	-
-	-	-	-	629.2797 ^d^ (−1.14)	-
C_24_H_34_O_9_	[T2GlcGlc–GlcGlc + H]^+^ ([T2 + H]^+^)	467.2276	N.D ^c^	467.2276 ^e^ (0.15)	N.D^c^
-	-	-	-	-	-
C_19_H_24_O_7_	[T2GlcGlc–GlcGlc–C_5_H_10_O_2_ + H]^+^	365.1595	N.D ^c^	365.1597 (0.51)	365.1593 (−0.40)
-	([T2–C_5_H_10_O_2_ + H]^+^ )	-	-	365.1596 ^d^ (0.34)	305.1597 ^d^ (0.60)
C_17_H_20_O_5_	[T2GlcGlc–GlcGlc–C_5_H_10_O_2_–C_2_H_4_O_2_ + H]^+^	305.1384	-	305.1383 (−0.15)	305.1383 (−0.25)
-	([T2–C_5_H_10_O_2_–C_2_H_4_O_2_ + H]^+^)	-	305.1361 ^d^ (−7.25)	305.1382 ^d^ (−0.54)	305.1382 ^d^ (−0.55)
C_15_H_16_O_3_	[T2GlcGlc–GlcGlc–C_5_H_10_O_2_–2C_2_H_4_O_2_ + H]^+^	245.1172	245.1170 (−0.82)	245.1179 (−0.14)	245.1172 ^e^ (−0.14)
-	([T2–C_5_H_10_O_2_–2C_2_H_4_O_2_ + H]^+^)	-	-	245.1169 ^d^ (−0.54)	245.1169 ^d^ (−1.44)
C_14_H_14_O_2_	[T2GlcGlc–GlcGlc–C_5_H_10_O_2_–2C_2_H_4_O_2_–CH_2_O + H]^+^	215.1067	215.1067 (0.30)	215.1066 (−0.20)	215.1068 (0.51)
-	([T2–C_5_H_10_O_2_–2C_2_H_4_O_2_–CH_2_O + H]^+^)	-	-	215.1064 ^d^ (−1.26)	215.1065 ^d^ (−0.56)

^a^ Mass value was calculated based on elemental formula; ^b^ Mass value detected with all ion MS/MS spectrum with collision energy (scan event 2) was shown with its parent compound name;^ c^ Not detected; ^d^ Mass value was detected by full scan (scan event 1); ^e^ Mass deviation was not apparent at this decimal representation level.

**Table 2:** Exact mass values of HT2GlcGlc and relative ions (calculated and observed) at negative polarity.

Elemental formula	Ion	Calculated mass ^a^	HT2GlcGlc (12.36 min)	HT2Glc (12.36 min)	HT2 (14.13 min)
			Observed mass ^b^ (mass acuracy, ppm)	Observed mass ^b^ (mass acuracy, ppm)	Observed mass ^b^ (mass acuracy, ppm)
C_36_H_56_O_20_	[HT2GlcGlc + CH_3_COO]^−^	807.3292	-	-	-
-	-	-	807.3296 ^d^ (0.46)	-	-
C_34_H_52_O_18_	[HT2GlcGlc − H]^−^	747.3081	747.3080 (−0.14)	-	-
C_28_H_42_O_13_	[HT2GlcGlc–Glc − H]^−^ ([HT2Glc − H]^−^)	585.2553	-	-	-
-	-	-	585.2551 ^d^ (−0.23)	585.2562 ^d^ (1.54)	-
C_22_H_32_O_8_	[HT2GlcGlc–GlcGlc − H]^−^ ([HT2 − H]^−^)	423.2024	N.D ^c^	423.2036 (2.77)	N.D ^c^
-	-	-	-	423.2025 ^d^ (0.17)	-
C_17_H_22_O_6_	[HT2GlcGlc–GlcGlc–C_5_H_10_O_2 _− H]^−^	321.1344	321.1354 (3.25)	321.1355 (3.44)	N.D^c^
-	([HT2–C_5_H_10_O_2_ − H]^−^ )	-	-	321.1352 ^d^ (2.49)	-
C_15_H_18_O_4_	[HT2GlcGlc–GlcGlc–C_5_H_10_O_2_–C_2_H_4_O_2_ − H]^−^	261.1132	261.1134 (0.54)	261.1135 (1.12)	261.1130 (−0.98)
-	([HT2–C_5_H_10_O_2_–C_2_H_4_O_2_ − H]^−^)	-	261.1345 ^d^ (0.61)	261.1137 ^d^ (1.71)	-
C_14_H_16_O_3_	[HT2GlcGlc–GlcGlc–C_5_H_10_O_2_–C_2_H_4_O_2_–CH_2_O − H]^−^	231.1027	231.1028 (0.57)	231.1030 (1.29)	231.1022 (−2.01)
-	([HT2–C_5_H_10_O_2_–C_2_H_4_O_2_–CH_2_O − H]^−^)	-	231.1024 ^d^ (−1.22)	231.1027 ^d,e^ (0.04)	231.1021 ^d^ (−2.60)

^a^ Mass value was calculated based on elemental formula; ^b^ Mass value detected with all ion MS/MS spectrum with collision energy (scan event 2) was shown with its parent compound name; ^c^ Not detected; ^d^ Mass value was detected by full scan (scan event 1); ^e^ Mass deviation was not apparent at this decimal representation level.

**Table 3:** Exact mass values of MASGlc and relative ions (calculated and observed) at negative polarity.

Elemental formula	Ion	Calculated mass ^a^	MASGlc (10.65 min)	MAS (11.53 min)
			Observed mass ^b^ (mass acuracy, ppm)	Observed mass ^b^ (mass acuracy, ppm)
C_25_H_38_O_13_	[MASGlc + CH_3_COO]^−^	545.2240	-	-
-	-	-	545.2246 ^d^ (1.07)	-
C_23_H_34_O_11_	[MASGlc − H]^−^	485.2028	485.2034 (1.10)	N.D ^c^
-	-	-	485.2036 ^d^ (1.60)	-
C_17_H_24_O_6_	[MASGlc–Glc − H]^− ^([MAS − H]^−^)	323.1500	323.1500 ^e^ (−0.06)	N.D ^c^
-	-	-	323.1509 ^d^ (2.68)	-
C_15_H_18_O_4_	[MASGlc–Glc–C_2_H_4_O_2 _− H]^−^	261.1132	261.1134 (0.54)	261.1134 (0.54)
-	([MAS–C_2_H_4_O_2 _− H]^−^)	-	261.1134 ^d^ (0.65)	261.1131 ^d^ (−0.63)
C_14_H_16_O_3_	[MASGlc–Glc–C_2_H_4_O_2_–CH_2_O − H]^−^	231.1027	231.1024 (−1.35)	231.1028 (0.63)
-	([MAS–C_2_H_4_O_2_–CH_2_O − H]^−^)	-	231.1023 ^d^ (−1.48)	231.1027 ^d^^,e^ (0.24)

^a^ Mass value was calculated based on elemental formula; ^b^ Mass value detected with all ion MS/MS spectrum with collision energy (scan event 2) was shown with its parent compound name; ^c^ Not detected; ^d^ Mass value was detected by full scan (scan event 1); ^e^ Mass deviation was not apparent at this decimal representation level.

## 3. Results

### 3.1. Detection and Identification of T2GlcGlc

**Figure 2:**
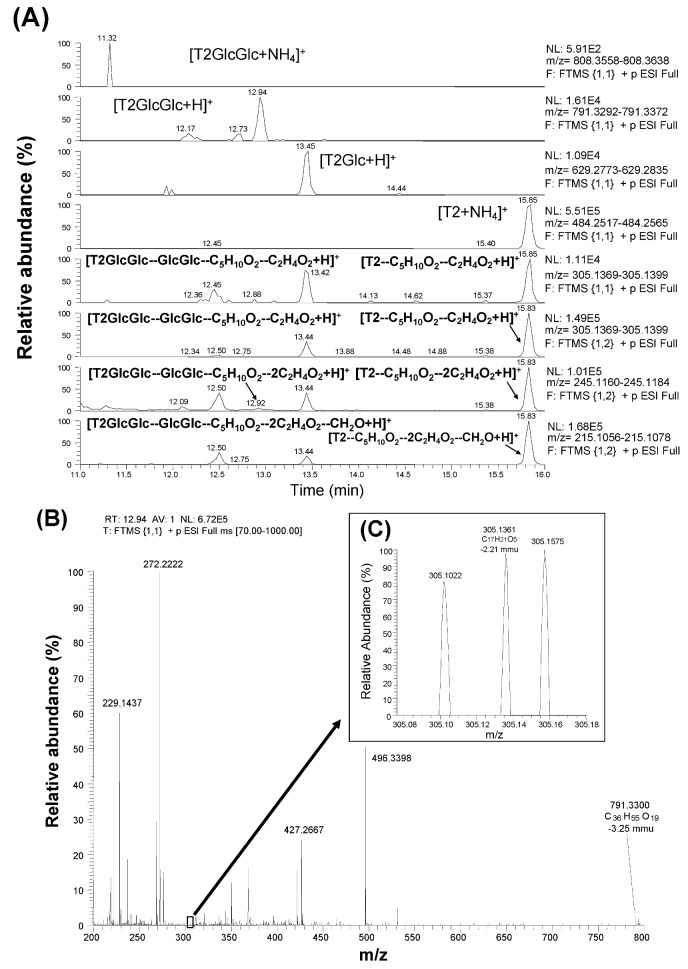
Detection and identification of T2GlcGlc by LC-Orbitrap MS with scan results (scan event 1 and 2) (**A**) and full MS spectrum obtained at 12.94 min (scan event 1) (**B**).

In the former study [15], neither ion of [T2GlcGlc + NH_4_]^+^ (808.3598) nor [HT2GlcGlc + NH_4_]^+^ (766.3492) was detected in the search for T2GlcGlc and HT2GlcGlc in the corn powder sample. Therefore, the authors searched for T2GlcGlc based on the [T2GlcGlc + H]^+^ ion in this study. When full scan results (scan event 1) were scrutinized with the calculated mass of [T2GlcGlc + H]^+^ (791.3332), three peaks were detected (at 12.17, 12.73, and 12.94 min), and the most abundant was at 12.94 min (Figure 2A). When full scan results were extracted with the calculated mass values of [T2Glc + H]^+^ (629.2804) (Table 1) and [T2 + NH_4_]^+^ (484.2541), peaks corresponding to T2Glc and T2 were detected at 13.45 min and 15.85, respectively (Figure 2A). Based on the loss of isovaleric acid, acetic acid, and folmaldehyde from the T2 structure, the calculated mass values of [T2GlcGlc–GlcGlc–C_5_H_10_O_2_ + H]^+^ (365.1595), [T2GlcGlc–GlcGlc–C_5_H_10_O_2_–C_2_H_4_O_2_ + H]^+^ (305.1384), [T2GlcGlc–GlcGlc–C_5_H_10_O_2_–2C_2_H_4_O_2_ + H]^+^ (245.1172), and [T2GlGlc–GlcGlc–C_5_H_10_O_2_–2C_2_H_4_O_2_–CH_2_O + H]^+^ (215.1067) ions (Table 1) were used when screening the result of both scan (scan event 1 and 2). Consequently, peaks at 12.92, 13.44, and 15.83 min were detected for the monitor ion of [T2GlcGlc–GlcGlc–C_5_H_10_O_2_–2C_2_H_4_O_2_ + H]^+^ (245.1172) during scan event 2 (shown in the second lowest column in Figure 2A), suggesting that similar fragmentation was occurring with T2GlcGlc, T2Glc, and T2. Figure 2B shows a full scan mass spectrum extracted at 12.94 min. The mass values of parental [T2GlcGlc + H]^+^ (791.3300) and typical fragment [T2GlcGlc–GlcGlc–C_5_H_10_O_2_–C_2_H_4_O_2_ + H]^+^ (305.1361) were observed with the mass deviation values of −3.25 mmu (−4.11 ppm) and −2.21 mmu (−7.25 ppm), respectively. This fragment ion (Figure 2C) was recognized only when magnifying the spectra due to the low intensity of the signal. The observed mass ions at each scan event and their respective mass deviations from the calculated values are summarized in Table 1. The earlier elution of T2GlcGlc (12.94 min) from C_18_ column compared with T2Glc (13.45 min) and T2 (15.85 min) suggests that T2GlcGlc is more hydrophilic than T2Glc and T2. Considering the above data, the compound detected at 12.94 min is T2GlcGlc. With regard to the other two peaks corresponding to the mass value of [T2GlcGlc + H]^+^ at 12.17 min and 12.73 min (Figure 2A), ions with mass values of 791.3326 and 791.3317 were detected with reasonable deviation (−2.10 and −3.33 ppm, respectively). Therefore, these two peaks were suggested to be those of T2GlcGlc isomers.

### 3.2. Detection and Identification of HT2GlcGlc

**Figure 3:**
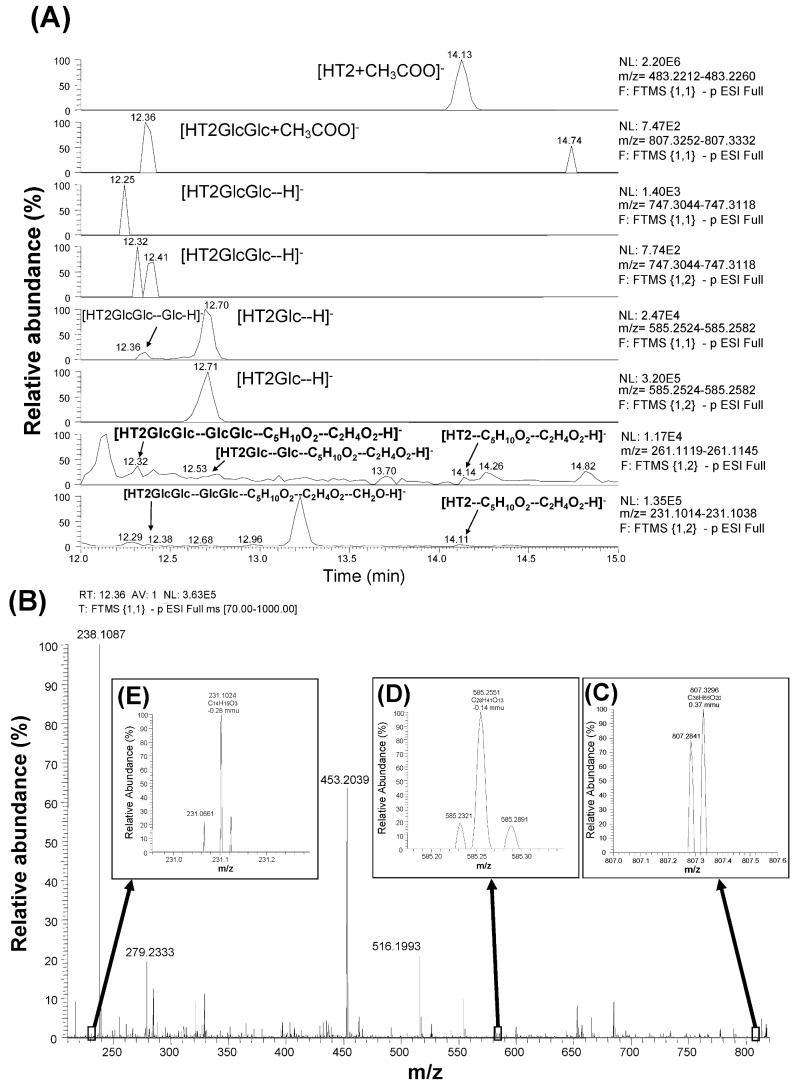
Detection and identification of HT2GlcGlc by LC-Orbitrap MS with scan results (scan event 1 and 2) (**A**) and full MS spectrum obtained at 12.36 min (scan event 1) (**B**).

Figure 3 shows the results of screening for HT2GlcGlc. When the authors searched for HT2GlcGlc at the positive polarity based on the calculated mass value of [HT2GlcGlc + H]^+^ (766.3491), clear peak supposed to be HT2GlcGlc was not apparent in the former study [15]. On the other hand, HT2 was found to be detected at the negative polarity in the form of [HT2 + CH_3_COO]^−^ at 14.13 min as shown at the top of Figure 3A, which was confirmed with the HT2 standard. Therefore, HT2GlcGlc was searched at the negative polarity in this study. When full scan results (scan event 1) was scrutinized with the calculated mass values of [HTGlcGlc + CH_3_COO]^−^ (807.3292) and [HTGlcGlc − H]^−^ (747.3081), a peak consistent with the former was detected at 12.36 min (Figure 3A). When full scan results (scan event 1) were screened for an ion with a calculated mass corresponding to [HT2GlcGlc–Glc − H]^−^ (585.2553), two peaks were detected at 12.36 and 12.70 min (Figure 3A), and the latter was consistent with HT2Glc previously reported [15]. Based on the loss of isovaleric acid, acetic acid, and formaldehyde from the HT2 structure, the calculated mass values of [HT2GlcGlc–GlcGlc–C_5_H_12_O_2_ − H]^−^ (321.1334), [HT2GlcGlc–GlcGlc–C_5_H_12_O_2_–C_2_H_4_O_2_ − H]^−^ (261.1132), and [HT2Glc–GlcGlc–C_5_H_12_O_2_–C_2_H_4_O_2_–CH_2_O − H]^−^ (231.1027) ions (Table 2), were used for the screening the result of each scan. Consequently, peaks at 12.38, 12.68, and 14.11 min were detected for the monitor ion of fragments of [HT2Glc–GlcGlc–C_5_H_12_O_2_–C_2_H_4_O_2_–CH_2_O − H]^−^ (231.1027) during scan event 2 (shown at the bottom of Figure 3A), suggesting that similar fragmentation was occurring with HT2GlcGlc, HT2Glc, and HT2. Figure 3B shows a full scan mass spectrum extracted at 12.36 min. The mass values of parental [HTGlcGlc + CH_3_COO]^−^ (807.3296) (Figure 3C), and typical fragments of [HT2GlcGlc–Glc − H]^−^ (585.2551) (Figure 3D) and [HT2Glc–GlcGlc–C_5_H_12_O_2_–C_2_H_4_O_2_–CH_2_O − H]^−^ (231.1024) (Figure 3E) were observed with the mass deviation values of 0.37 mmu (0.46 ppm), −0.14 mmu (−0.23 ppm), and −0.28 mmu (−1.22 ppm), respectively. These ions were recognized only when magnifying the spectra due to the low intensity of the signal. The earlier elution of HT2GlcGlc (12.36 min) from C_18_ column compared with HT2Glc (12.70 min) and HT2 (14.13 min) suggests that HT2GlcGlc is more hydrophilic than HT2Glc and HT2. Considering the above data, the compound detected at 12.36 min is HT2GlcGlc. Since the low intensity of HT2GlcGlc (7.42 × E2) (observed in the second column in Figure 3A), other peaks concerned with its isomers were not observed. With regard to the existence of tri-glucosides, neither the ion of [T2GlcGlcGlc + H]^+^ nor [HT2GlcGlcGlc + CH3COO]^−^ was detected in the search for T2GlcGlcGlc or HT2GlcGlcGlc under these conditions.

### 3.3. Detection and Identification of MASGlc

**Figure 4:**
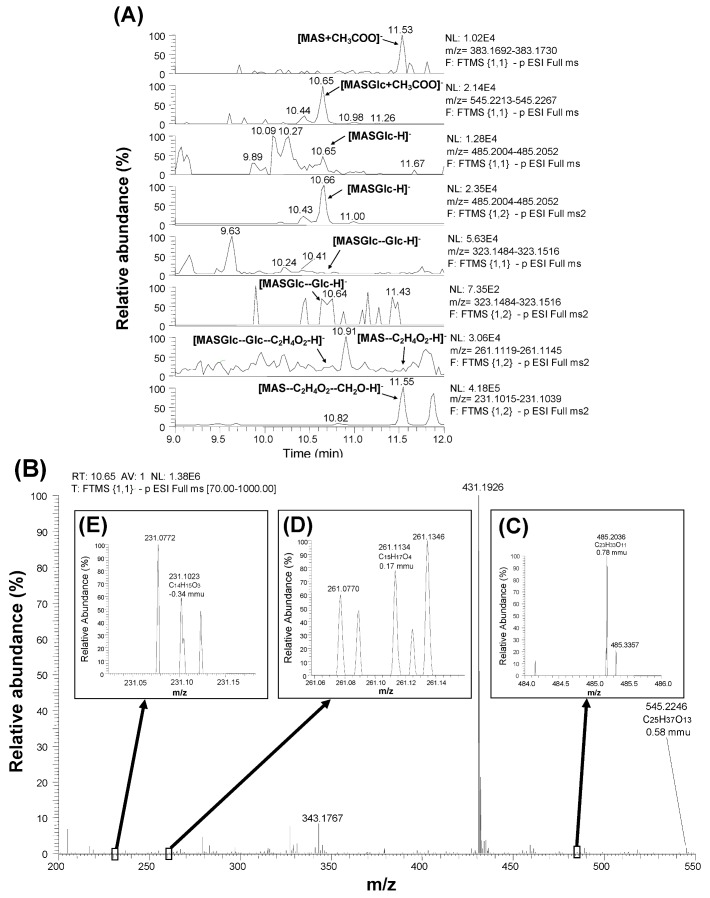
Detection and identification of MASGlc by LC-Orbitrap MS with scan results (scan event 1 and 2) (**A**) and full MS spectrum obtained at 10.65 min (scan event 1) (**B**).

When full scan results (scan event 1) were scrutinized with the calculated mass value of [MASGlc + H]^+^ (487.2174) and [MASGlc + NH_4_]^+^ (504.2439), a peak corresponding to [MASGlc + NH_4_]^+^ was slightly detected [21]. However, the signals with [MAS + H]^+^ (325.1646) and [MAS + NH_4_]^+^ (342.1911) were hardly detected, and further confirmation was not conducted. In this study, MAS was found to be detected at 11.53 min at negative polarity in the form of [MAS + CH_3_COO]^−^ as shown at the top of Figure 4A, which was confirmed with the MAS standard. Therefore, MASGlc was searched at negative polarity. When scan results (scan events 1 and 2) were scrutinized with the calculated mass values of [MASGlc + CH_3_COO]^−^ (545.2240) and [MASGlc − H]^−^ (485.2028), two peaks were detected at 10.65–66 and 10.43–44 min, and the former one was abundant (Figure 4A). Figure 4B shows a full scan mass spectrum extracted at 10.65 min. The mass values of parental [MASGlc + CH_3_COO]^−^ (545.2246) and [MASGlc − H]^−^ (485.2036) (Figure 4C) were observed with the mass deviation values of 0.58 mmu (1.07 ppm) and 0.78 mmu (1.60 ppm), respectively. Based on the loss of acetic acid and formaldehyde from the MAS structure, the calculated mass values of [MASGlc–Glc − H]^−^ (323.1500), [MASGlc–Glc–C_2_H_4_O_2 _− H]^−^ (261.1132), and [MASGlc–Glc–C_2_H_4_O_2_–CH_2_O − H]^−^ (231.1027) ions (Table 3) were used when screening the result of both scan (scan events 1 and 2). Consequently, a peak at 10.64 min was apparent for the monitor ion of [MASGlc–Glc − H]^−^ (323.1500) during scan event 2 (Figure 4A), suggesting that MAS fragment is detected at this retention time. In addition, typical fragments of [MASGlc–Glc–C_2_H_4_O_2_ − H]^−^ (261.1134) and [MASGlc–Glc–C_2_H_4_O_2_–CH_2_O − H]^−^ (231.1023) were observed with the mass deviation values of 0.17 mmu (0.65 ppm) and −0.34 mmu (−1.48 ppm) (Figure 4D,E). These ions were recognized only when magnifying the spectra due to the low intensity of the signal. The earlier elution of MASGlc (10.65 min) from C_18_ column compared with MAS (11.53 min) suggests that MASGlc is more hydrophilic than MAS. Considering the above data, the compound detected at 10.65 min is MASGlc, and another peak detected at 10.44 min is possibly suggested to be its isomer like another MASGlc previously reported [22].

## 4. Discussion

Authors previously reported the existence of mono-glucosylated derivatives of T2 and HT2 in the corn powder [14,15]. However, more highly glucosylated derivatives such as T2GlcGlc and HT2GlcGlc were not found at that time through the screening based on the calculated mass values of [T2GlcGlc + NH_4_]^+^ (808.3598) and [HT2GlcGlc + NH_4_]^+^ (766.3491). Certainly, T2, HT2, and their mono-glucosylated derivatives (T2Glc and HT2Glc) were well detected in the form of the ammonium adduct ions ([T2Glc + NH_4_]^+^ and [HT2Glc + NH_4_]^+^) rather than proton adduct ions ([T2Glc + H]^+^ and [T2Glc + H]^+^), and the ion intensity of the ammonium adducts was higher than that of the proton adducts [15]. However in this study, it was found that T2GlcGlc was detected as [T2GlcGlc + H]^+^ and HT2GlcGlc was detected as [HT2GlcGlc + CH_3_COO]^−^ (Figure 2, Figure 3). This is probably because the chemical properties of T2 and HT2 forming ammonium adduct was diminished as the number of glucose molecules conjugated to them was increased. The formation of ammonium adduct is usually observed only with the type A trichothecenes, and not observed with the type B trichothecenes. Probably some specific residue(s) in the type A trichothecene structure may result in the nucleophilic property forming the ammonium adduct ion. However, such nucleophilic property of the type A trichothecenes seem to be gradually replaced with that of glucose (not nucleophilic) as the number of the conjugated glucose is increased. In addition, some type A trichothecenes such as HT2 and MAS were detected at negative polarity in the form of acetic acid adduct ion ([M + CH_3_COO]^−^), indicating that the negative polarity was sometimes effective for detecting their respective glucosides (HT2GlcGlc and MASGlc). 

Zachariasova *et al*. reported the existence of DON-di-glucoside and DON-tri-glucosides in a beer sample containing DON [23]. They suggested the isometric formations for these glucosides since several peaks of the same molecular weight were observed at different retention time. Based on the structural similarity to DON, such isometric formation of di-glucosides could be suggested for the other trichothecenes. In the former study [13], the authors suggested the possibility that a kind of “masked mycotoxin cluster” (composed of mycotoxin mono-, di-, tri-, and poly-glucosides) can be produced, and this suggestion was supported by the above data. Veprikova *et al.* detected HT2GlcGlc in two independent barley samples through the screening on 20 *Fusarium*-infected samples of barley, wheat and oats [17]. In our study, similar peaks as observed with Figure 2A were also detected when another corn powder material (batch number TC-973) from Trilogy Co. Ltd was analyzed [21] indicating that T2GlcGlc could be found in other than the single lot sample (batch number MTC-9999D) used in this study. Although the existence of tri-glucosides was not confirmed in this study, such glucosides are possibly detected if they are formed at some concentration level. Kostelanska *et al*. [24] suggested that DON-oligo-glucosides are contained in fermented intermediates as well as in final products such as bread and malt/beer. As the origin of these DON-oligo-glucosides, they suggested that the splitting of alpha-glycosidic bonds between DON-3-glucoside and cell polysaccharides was occurring within brewing and baking technologies [24]. If such alpha-glycosidic bonds are formed in the corn with the type A trichothecene glucosides reported here (T2Glc, HT2Gla, and MASGlc), their amounts as well as those of respective di-glucoside derivatives may also be increased with the treatment of some glycosidases. These findings bring us to recognize that the existence of masked mycotoxins as a potential risk may not be negligible, since they could be transformed to the corresponding aglycons under certain conditions, for example treatment with several intestinal lactic acid bacteria [12], and/or in the digestive tract of swine after ingestion [10]. 

**Figure 5:**
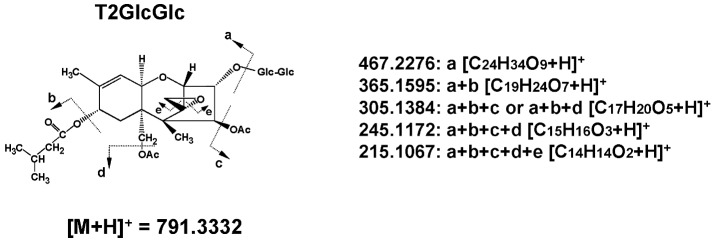
Proposed structures of T2GlcGlc and its possible fragments during ionization.

LC-Orbitrap MS is a special type of ion trap [25], and achieves a mass resolving power of up to 100,000 FWHM (*m/z* 200) and maintains mass accuracy (<5 ppm) even without the use of continuous internal mass correction. Therefore, it can detect and identify the new *Fusarium* masked mycotoxins whose chemical standards are not available. Alternatively, the absolute structure is not determined with LC-Orbitrap MS, since the structural isomers of the same elemental composition are often difficult to be distinguished. In the former studies, the absolute structure of T2Glc was fixed as T2-3-glucoside because there was only one OH residue in T2 molecule [15]. However, in the case of T2GlcGlc, the absolute structure was not fixed because the position of second glucose conjugation to T2Glc, which has four OH residues in its molecule (Figure 1) was not specified. Considering the structural hindrance in T2Glc molecule, glucosylation at 6'-OH residue of glucose branch seems to be most probable, whereas another possibility of glucosylation at 3'-OH or 4'-OH of glucose branch seems to be still remaining. Alternatively, 2'-OH residue seems to be difficult to be glucosylated due to the structural hindrance. The proposed structure of T2GlcGlc and its possible fragments are shown in Figure 5. Due to the lack of information on the position of the second glucose conjugation to T2Glc, the di-glucoside residue is represented as “-GlcGlc” here. Three peaks were observed with the mass value of [T2GlcGlc + H]^+^ at different retention time (12.17 min, 12.73 min, and 12.94 min) (Figure 2A). These peaks indicate the formation of such isomers, as observed with DON-di-glucoside detection by LC-Orbitrap MS [23]. In the case of HT2GlcGlc, the specification of the structure seems to be more complicated because HT2 has two possible positions (at 3-OH and 4-OH) to be glusocylated. Lattanzio *et al.* also detected T2Glc and HT2Glc in wheat and oats [16]. They especially detected two HT2Glc isomers, namely HT2-3-glucoside and HT2-4-glucoside with a monitor ion of [HT2Glc + NH_4_]^+^ [16], whereas the latter was not observed by the authors in the previous study, probably due to minority in the amount [15]. The assumption that glucosylation of HT2 mainly occurs at C-3 position rather than at C-4 in corn corresponds to the fact that T2 having only one OH at C-3 position was concurrently glucosylated [15,18]. Alternatively, two peaks were detected for the screening of MASGlc with the monitor ion of [MASGlc + CH_3_COO]^−^ at different retention time (10.65 and 10.44 min) (Figure 4A). Based on the above consideration, MAS3Glc is suggested to correspond to the larger peak (10.65 min) and MAS4Glc to the smaller one (10.44 min). The latter is reported to be produced by *Fusarium sambucinum* [22]. 

Another arising question may be concerned with the amounts of these glucosides. However, it was not possible to measure their amounts due to the lack of appropriate chemical standards. From the calibrations using standard solutions, the concentrations of T2, HT2, MAS, and DON in the corn sample were determined to be 184, 304, 7.8, and 1780 μg kg^−^^1^, respectively. Compared with the manufacturer-labeled concentrations of the toxins (300 ± 57 μg kg^−^^1^ for T2, 510 ± 83 μg kg^−^^1^ for HT2, and 2200 ± 600 μg kg^−^^1^ for DON), the extraction efficiency of T2, HT2, and DON from the corn powder sample was estimated to be 52%–76% (T2), 51%–71% (HT2), and 64%–111% (DON), respectively. The availability of analytical standards should be essential to promote the works concerned with masked mycotoxins. In addition, the conformation studies in terms of the anomeric structures of alpha- and beta- isomers on these glucosides have not been developed well. Such information may also be obtained in accordance with the NMR analysis if sufficient amount of standards are supplied.

## 5. Conclusions

The existence of di-glucosylated derivatives of T2 in plant was confirmed for the first time by means of LC-Orbitrap MS. Natural contamination of corn with di-glucosylated derivative of HT2 and mono-glucoside derivative of MAS was also discovered in this study. The novelty of the technique in the present study as compared with the prior studies [15,21] is that we focused on the proton adduct [M + H]^+^ rather than the ammonium adduct [M + NH_4_]^+^ through the screening of T2GlcGlc. Another challenge was the use of the negative scan mode that was usually used for the detection of type B trichothecenes for the detection of HT2GlcGlc and MASGlc. Although the existence of these masked mycotoxins is not counted with the risk evaluation, more analytical and toxicological studies on them are needed to obtain more information on the prevalence in foods and their relevance for human health.
